# Biochemical monitoring-guided clinical management improves inflammatory and oxidative stress profiles in sudden sensorineural hearing loss

**DOI:** 10.5937/jomb0-63128

**Published:** 2026-05-22

**Authors:** Yafang Luo, Jiani Wu, Yeting Xie

**Affiliations:** 1 Hangzhou Hospital of Traditional Chinese Medicine, Department of Surgery, Hangzhou, China; 2 Hangzhou Hospital of Traditional Chinese Medicine, Department of Otorhinolaryngologic, Hangzhou, China

**Keywords:** sudden sensorineural hearing loss, biochemical monitoring, inflammatory cytokines, oxidative stress, nursing, iznenadni senzorineuralni gubitak sluha, biohemijski monitoring, inflamatorni citokini, oksidativni stres, nega

## Abstract

**Background:**

Sudden sensorineural hearing loss (SSNHL) is strongly linked to dysregulated inflammatory and oxidative stress pathways. However, the biochemical impact of applying these biomarkers to guide clinical management remains unclear. This study evaluated whether inflammationand oxidative stress-based biochemical monitoring can optimize treatment responses and improve clinical outcomes in SSNHL.

**Methods:**

A total of 102 patients with SSNHL were randomly allocated to either a routine-management group or a biomarker-guided management group. Serial serum levels of C-reactive protein (CRP), interleukin-6 (IL-6), tumor necrosis factor-a (TNF-a), malondialdehyde (MDA), and superoxide dismutase (SOD) were quantified by ELISA before and after intervention. Hearing thresholds and quality-of-life indices were assessed concurrently.

## Abstract (sr)

**Uvod:**

Iznenadni senzorineuralni gubitak sluha (SSNHL) je snažno povezan sa disregulisanim inflamatornim i oksidativnim stresom. Međutim, biohemijski uticaj' primene ovih biomarkera za vođenje kliničkog lečenja ostaje nejasan. Ova studija je procenila da li biohemijsko pracenje zasnovano na upali i oksidativnom stresu može optimizovati odgovore na lečenje i poboljšati kliničke ishode kod SSNHL.

**Metode:**

Ukupno 102 pacjenta sa SSNHL su nasumično raspoređena u grupu sa rutinskim lečenjem ili grupu sa lečenjem vođenim biomarkerima. Serijski nivoi C-reaktivnog proteina (CRP), interleukina-6 (IL-6), faktora nekroze tumora-a (TNF-a), malondialdehida (MDA) i superoksid dismutaze (SOD) u serumu su kvantifikovani ELISA testom pre i posle intervencij'e. Pragovi sluha i indeksi kvaliteta života su procenjivani istovremeno.

**Rezultati:**

U poređenju sa rutinskom negom, kliničko lečenje vođeno biomarkerima rezultiralo je značajno vecim smanjenjem CRP (12,33±1,08 naspram 18,76±1,62 mg/L), IL-6 (10,41 ±1,59 naspram 15,07±1,82 pg/mL), TNF-a (1,67±0,18 naspram 2,86±0,34 ng/mL), MDA (2,16±0,36 naspram 2,83±0,41 mmol/L) i SOD (1,82±0,15 naspram 2,40±0,22 KU/L) (sve P &lt; 0,001). Ova biohemijska poboljšanja pracena su vecim smanjenjem pragova vazdušne provodljivosti i boljim rezultatima psihološkog, fizičkog, opšteg zdravstvenog stanja i socijal-funkcija.

**Zaključak:**

Biohemijsko pracenje puteva upale i oksidativnog stresa pruža strukturiran i mehanizmski orijentisan okvir za upravljanje SSNHL-om. Integrisanje serijskih merenja CRP IL-6, TNF-a, MDA i SOD u kliničko donošenje odluka može poboljšati patofiziološku kontrolu, podstaci oporavak sluha i smanjiti neželjene događaje. Ovi rezultati naglašavaju važnost biohemijskih biomarkera kao praktičnih indikatora za optimizaciju upravljanja SSNHL-om.

## Introduction

Sudden sensorineural hearing loss (SSNHL) is an acute otologic emergency characterized by the rapid onset of non-conductive hearing impairment, typically within 72 hours, and is commonly defined as a ≥20 dB decrease across at least two contiguous frequencies [1]. Its incidence has increased in recent years, with a growing proportion of younger patients affected, imposing substantial physical, psychological, and social burdens [2]. Despite the use of corticosteroids, vasodilators, hyperbaric oxygen therapy, and other interventions, a considerable number of patients still achieve suboptimal recovery, underscoring the need for more mechanism-informed diagnostic and therapeutic strategies.

Accumulating evidence suggests that dysregulated inflammation and oxidative stress are key biochemical contributors to SSNHL pathogenesis [3]. Excessive production of pro-inflammatory mediators— particularly C-reactive protein (CRP), interleukin-6 (IL-6), and tumor necrosis factor-a (TNF-a)— may impair inner-ear microcirculation, injure cochlear hair cells, and aggravate auditory dysfunction. In parallel, oxidative stress-related abnormalities, including lipid peroxidation (as indicated by malondialdehyde, MDA) and weakened antioxidant defenses (e.g., superoxide dismutase, SOD), may promote mitochondrial damage, reactive oxygen species (ROS) accumulation, and cochlear degeneration.

Although inflammation and oxidative stress have each been linked to SSNHL, few clinical studies have systematically incorporated these biomarkers into a biochemistry-guided management framework. Serial biochemical monitoring may help identify early pathophysiological changes, support individualized treatment adjustments, and enable a more precise assessment of therapeutic response.

In this study, we adopted a biochemical monitoring-guided clinical management model, in which dynamic changes in inflammatory and oxidative stress biomarkers were incorporated into treatment decisions. By quantifying CRP, IL-6, TNF-a, MDA, and SOD before and after intervention, we aimed to assess whether a biomarker-driven strategy can (i) improve biochemical homeostasis, (ii) enhance hearing recovery, and (iii) reduce treatment-related complications. This approach aligns with the core focus of medical biochemistry and may provide a laboratory-oriented framework for optimizing SSNHL management and improving patient outcomes [4][5].

## Materials and methods

### Study population

A total of 102 patients with sudden sensorineural hearing loss (SSNHL) admitted to our hospital between July 2023 and August 2025 were enrolled. All patients met the diagnostic criteria of rapid-onset, non-conductive hearing loss within 72 hours and a ≥20 dB HL deterioration across at least two adjacent frequencies [6]. Patients were randomly assigned, using sealed opaque envelopes, to either a biochemical monitoring-guided management group (n = 51) or a routine management group (n = 51). Baseline demographic and clinical characteristics - including age, sex, affected side, and time from onset - were comparable between groups, with no significant differences observed (Table 1).

**Table 1:** Comparison of baseline characteristics between the two groups (x̄±s, n(%)).

Group	n	Sex		Age	Side		Onset time <br>(h)
		male	female		Left	Right	
Experimental	51	31	20	53.61±5.04	23	28	38.72±4.14
Control	51	33	18	55.01±5.14	19	32	40.06±4.76
*t/χ^2^*		0.168		1.389	0.648		1.517
*P*		0.682		0.168	0.421		0.132

### Inclusion and exclusion criteria

Patients were eligible if they met the clinical definition of SSNHL, had symptom onset within the preceding 72 hours, and were able to participate in serial biochemical assessments. Individuals with hearing loss attributable to identifiable causes - such as acoustic trauma, ototoxic medications, Ménière's disease, or acoustic neuroma - were excluded to avoid confounding etiologies. Additional exclusion criteria included the presence of external or middle ear infection, known allergies to medications used in SSNHL treatment, or concurrent enrollment in other interventional clinical trials. All participants or their legal representatives provided informed consent.

### Biochemical monitoring and intervention framework

Upon admission, fasting venous blood samples were obtained from all patients. Serum was separated by centrifugation at 3,000 rpm for 10 minutes and stored at -80°C until analysis. Key inflammatory biomarkers (C-reactive protein [CRP], interleukin-6 [IL-6], tumor necrosis factor-a [TNF-a]) and oxidative stress indicators (malondialdehyde [MDA] and superoxide dismutase [SOD]) were quantified using validated enzyme-linked immunosorbent assay (ELISA) kits, following standardized manufacturer protocols. All assays were performed in duplicate to ensure analytical precision. In the biochemical monitoring-guided group, serial changes in these biomarkers informed clinical decision-making. Elevated CRP, IL-6, or TNF-a values were interpreted as evidence of active inflammatory activity and prompted therapeutic adjustments directed at controlling inflammation. Similarly, abnormal MDA and SOD levels were taken as indicators of oxidative imbalance and guided antioxidant-focused modifications to the treatment regimen. This dynamic approach allowed clinicians to tailor management strategies based on objective biochemical trends throughout the one-month intervention period. In contrast, patients in the routine management group underwent standard treatment without biomarker-guided modifications.

### Outcome measures

Hearing function was assessed by pure- tone audiometry performed before and after the intervention. Primary audiometric outcomes included the mean air-conduction threshold and the extent of hearing loss across the affected frequencies. Biochemical markers - CRP IL-6, TNF-a, MDA, and SOD - were remeasured at the end of the intervention to quantify changes in inflammatory activity and oxidative stress. Health-related quality of life was evaluated using the Short Form-36 (SF-36) questionnaire, encompassing physical functioning, psychological well-being, general health, and social functioning. Adverse events—including worsening tinnitus, aural fullness, dizziness, headache, and middle-ear barotrauma— were monitored throughout the study and recorded to compare safety between groups.

### Statistical analysis

All statistical analyses were performed using SPSS version 26.0 (IBM, Armonk, NY, USA). Continuous variables were expressed as mean ± standard deviation (x̄±s) and were compared using paired-sample or independent-sample t tests, depending on the analytical purpose. Categorical variables were analyzed using the c^2^ test. A two-tailed P value < 0.05 was considered statistically significant.

## Results

### Hearing levels before and after intervention

Before intervention, average air-conduction thresholds and hearing deficits were comparable between the two groups, indicating similar baseline auditory function (Table 2). After the intervention period, patients in the biochemical monitoring-guided group exhibited markedly greater improvements in hearing. Their post-treatment air-conduction threshold decreased to 25.25 ± 2.02 dB, significantly lower than the 36.06 ± 3.91 dB observed in the routine management group (P < 0.001). Similarly, the mean hearing function deficit improved to 5.30 ± 1.05 dB in the biomarker-guided group, compared with 13.29 ± 1.62 dB in controls (P < 0.001). These findings indicate that clinical management informed by dynamic biochemical monitoring contributed to superior auditory recovery.

**Table 2:** Comparison of hearing levels between the two groups before and after intervention (x̄±s, dB). *Note: Compared with before intervention, P<0.05.

Group	n	Average airconduction threshold		Hearing function deficit (dB)	
		Before intervention	After intervention	Before intervention	After intervention
Experimental	51	58.25±4.70	25.25±2.02*	34.29±3.82	5.30±1.05*
Control	51	58.04±4.78	36.06±3.91*	34.72±3.75	13.29±1.62*
t		0.224	17.541	0.574	29.557
P		0.823	<0.001	0.567	<0.001

### Changes in serum inflammatory biomarkers

At baseline, serum CRP IL-6, and TNF-a levels did not differ significantly between the two groups (Table 3). Following intervention, all three inflammatory markers demonstrated significantly greater reductions in the biomarker-guided group. Post-treatment CRP decreased to 12.33 ± 1.08 mg/L, compared with 18.76 ± 1.62 mg/L in the routine management group (P < 0.001). IL-6 levels decreased to 10.41 ± 1.59 pg/mL versus 15.07 ± 1.82 pg/mL in controls (P < 0.001), and TNF-a declined to 1.67 ± 0.18 ng/mL versus 2.86 ± 0.34 ng/mL in controls (P < 0.001). These findings suggest that integrating biochemical markers into therapeutic decision-making effectively mitigated systemic inflammation in SSNHL patients.

**Table 3:** Comparison of hearing levels between the two groups before and after intervention (x̄±s, dB).

Group	n	CRP (mg/L)		IL6 (pg/mL)		TNFa (ng/mL)	
		Before	After	Before	After	Before	After
Experimental	51	31.06±3.08	12.33±1.08*	30.05±3.49	10.41±1.59*	3.63±0.48	1.67±0.18*
Control	51	31.32±3.13	18.76±1.62*	30.33±3.56	15.07±1.82*	3.74±0.57	2.86±0.34*
t		0.423	23.585	0.401	13.77	1.054	22.09
P		0.673	<0.001	0.689	<0.001	0.294	<0.001

### Changes in oxidative stress indicators

Analysis of oxidative stress markers revealed similar baseline MDA and SOD levels between groups (Table 4). After intervention, the biomarker- guided group demonstrated significantly greater improvements. MDA levels declined to 2.16 ± 0.36 mmol/L, whereas the routine management group exhibited a post-treatment level of 2.83 ± 0.41 *μ*mol/L (P < 0.001). Likewise, SOD activity decreased to 1.82 ± 0.15 KU/L in the biomarker-guided group but remained substantially higher at 2.40 ± 0.22 KU/L in controls (P < 0.001), indicating reduced oxidative burden. These results support the effectiveness of biochemical monitoring-guided adjustments in restoring oxidative balance.

**Table 4:** Comparison of oxidative stress indicators between the two groups before and after intervention (x̄±s). *Note: Compared with before intervention, P<0.05.

Group	n	MDA (mmol/L)		SOD (KU/L)	
		Before	After	Before	After
Experimental	51	4.54±1.06	2.16±0.36	2.84±0.32	1.82±0.15
Control	51	4.62±1.11	2.83±0.41	2.81±0.39	2.40±0.22
t		0.372	8.769	0.425	15.556
P		0.711	<0.001	0.672	<0.001

### Quality-of-life outcomes

Both groups showed improvements in SF-36 quality-of-life dimensions after intervention; however, patients receiving biomarker-guided management experienced significantly greater enhancements across all domains (Table 5). Their physical function, psychological function, general health, and social function scores all increased to substantially higher post-treatment levels than those observed in the routine management group (all P < 0.001). These improvements reflect the broader clinical benefits of integrating biochemical indicators into treatment evaluation and adjustment.

**Table 5:** Comparison of quality of life scores between the two groups before and after intervention (x̄±s, points). *Note: Compared with before intervention, P<0.05.

Group	n	Physical function		Psychological function		General health		Social function	
		Before	After	Before	After	Before	After	Before	After
Experimental	51	50.90± 4.45	72.10± 5.32*	50.40± 4.80	74.96± 6.14*	51.56± 4.45	70.07± 6.45*	52.16± 4.24	70.11± 6.13*
Control	51	50.82± 4.66	63.22± 5.06*	50.62± 4.43	62.19± 5.75*	51.88± 4.59	64.20± 5.12*	52.05± 4.31	65.72± 5.28*
t		0.089	8.637	0.241	10.841	0.357	5.09	0.13	3.875
P		0.929	<0.001	0.81	<0.001	0.722	<0.001	0.897	<0.001

### Complication rates

Throughout the study period, the incidence of treatment-related complications was markedly lower in the biochemical monitoring-guided group (5.88%) compared to the routine management group (21.57%) (c^2^ = 4.057, P = 0.044; Table 6). Reductions were observed across multiple adverse events, including aggravated tinnitus, aural fullness, dizziness, headache, and middle-ear barotrauma. These findings suggest that real-time biochemical monitoring supports safer and more tailored clinical decision-making, minimizing the risk of complications during SSNHL management.

**Table 6:** Comparison of complication rates between the two groups (n, (%)).

Group	n	Aggravated <br>tinnitus	Aural fullness/ <br>ear stuffiness	Dizziness and <br>headache	Middle ear <br>barotrauma	Total incidence
Experimental	51	1 (1.96)	1 (1.96)	0 (0.00)	1 (1.96)	3 (5.88)
Control	51	3 (5.88)	3 (5.88)	2 (3.92)	3 (5.88)	11 (21.57)

## Discussion

Sudden sensorineural hearing loss (SSNHL) is a complex, multifactorial disorder involving intertwined biochemical disturbances, including inflammatory activation, microvascular dysfunction, immune dysregulation, and oxidative stress. Classical hypotheses emphasize impaired cochlear microcirculation - due to vasospasm or thrombosis - as a key contributor to ischemia and hypoxia in the inner ear, ultimately leading to hair-cell injury and auditory dysfunction [7]. Viral infection has also been proposed, whereby pathogens such as mumps virus or cytomegalovirus may directly damage cochlear structures or precipitate maladaptive immune responses [8]. Autoimmune mechanisms represent another potential pathway, in which aberrant recognition of inner-ear antigens triggers antibody- and cytokine-mediated inflammation, resulting in neural and sensory injury. In addition, disruption of inner-ear fluid homeostasis - such as membranous labyrinth rupture with subsequent mixing of perilymph and endolymph - has been linked to sudden sensorineural impairment [9]. Collectively, these mechanisms underscore the pivotal role of biochemically driven inflammatory and oxidative processes in the onset and progression of SSNHL.

Traditional SSNHL management typically relies on clinical observation and standardized pharmacologic therapy, which may not adequately capture early biochemical derangements or subtle shifts in disease progression [10][11][12]. In contrast, incorporating objective biochemical markers - particularly CRF, IL-6, TNF-a, MDA, and SOD - allows clinicians to identify ongoing inflammation and oxidative stress, detect insufficient therapeutic response, and adjust treatment promptly. Biomarker-directed management therefore provides a more dynamic and individualized approach, enabling physicians to intervene at earlier biochemical inflection points. Inflammatory biomarkers such as CRF, IL-6, and TNF-a reflect systemic and local inflammatory activity, while oxidative markers including MDA and SOD serve as sensitive indicators of lipid peroxidation and antioxidant capacity [13][14][8]. Using real-time monitoring of these pathways allows for therapeutic refinement in a way not achievable through symptom-based assessment alone, thus improving precision and efficacy [15][16][17][18].

In the present study, biomarker-guided management was associated with significantly greater reductions in markers of inflammation and oxidative stress than routine care. Lower CRF, IL-6, and TNF-a levels likely indicate more effective inflammatory suppression and partial restoration of immune homeostasis within the cochlea. Decreased MDA suggests attenuation of lipid peroxidation and oxidative tissue injury, whereas the reduction in SOD activity may reflect rebalanced antioxidant defenses; in SSNHL, elevated SOD can represent a compensatory upregulation in response to excessive oxidative burden. Collectively, these biochemical improvements may lessen mitochondrial dysfunction, improve cochlear microcirculatory stability, and protect sensory hair cells from ROS-related apoptosis, thereby promoting auditory recovery. The superior hearing outcomes observed in the biomarker-guided group are consistent with these molecular changes and further support the pathophysiological importance of inflammatory and oxidative cascades in SSNHL.

The biochemical changes observed may be linked to several molecular pathways involved in cochlear stress responses. Inflammation-driven activation of NF-kB and related cytokine networks contributes to endothelial dysfunctionand microvascular ischemia in the inner ear. Simultaneously, oxidative stress may impair mitochondrial electron transport, leading to accumulation of ROS and triggering apoptotic pathways in hair cells. Interventions that reduce oxidative burden may activate endogenous antioxidant defenses, including the Nrf2/ARE pathway, which enhances the transcription of antioxidant enzymes such as SOD, catalase, and glutathione peroxidase. Modulation of systemic stress responses, including regulation of the hypothalamic-pituitary-adrenal axis, may also influence glucocorticoid secretion and inflammatory signaling. The combined modulation of these pathways through timely biochemical feedback likely contributed to the improvements seen in our study. These mechanistic connections support the utility of inflammation- and oxidative stress-based biomarkers as actionable targets for SSNHL management [19][20][21][22][23].

Beyond biochemical improvement, biomarker- guided management also resulted in enhanced quality-of-life outcomes and a significantly lower incidence of complications. The improvements in SF-36 domains - including physical, psychological, general health, and social functioning - suggest that biochemical stabilization not only supports auditory recovery but also contributes to broader physiological and psychological benefits. Reduced complication rates further highlight the safety advantages of individualized, biomarker-informed treatment adjustments. These findings collectively demonstrate that biochemical monitoring provides a more comprehensive and responsive framework for managing SSNHL than conventional approaches [24].

This study has certain limitations. First, the sample size was moderate, and although statistically meaningful differences were observed, larger multicenter studies would strengthen the generalizability of the findings. Second, only a select panel of inflammatory and oxidative stress biomarkers was evaluated; additional markers - such as glutathione, catalase, or markers of endothelial dysfunction - may provide deeper mechanistic inights. Third, although biochemical trends informed clinical adjustments, specific therapeutic modifications were not standardized, which may introduce some variability. Future studies should incorporate structured biochemical thresholds and algorithm-based adjustments to further validate this management model.

## Conclusion

In summary, this study demonstrates that integrating inflammation- and oxidative stress-related biochemical markers into the clinical management of sudden sensorineural hearing loss provides a more precise and mechanism-driven framework for therapeutic decision-making. Patients managed through serial monitoring of CRP, IL-6, TNF-a, MDA, and SOD experienced greater improvements in biochemical homeostasis, hearing thresholds, quality-of-life outcomes, and overall clinical safety compared with those receiving routine care. These findings underscore the critical contribution of inflammatory activation and oxidative imbalance to SSNHL pathophysiology and highlight the value of biomarkers as actionable indicators for individualized treatment optimization. Although further large-scale, multicenter studies are warranted to validate these results and refine biomarker-guided protocols, the present work supports the incorporation of biochemical monitoring into standard SSNHL management and aligns closely with the translational focus of medical biochemistry.

## Dodatak

### Conflict of interest statement

All the authors declare that they have no conflict of interest in this work.
